# The Importance of Secondary Gain - a Missing Story

**DOI:** 10.1192/j.eurpsy.2022.155

**Published:** 2022-09-01

**Authors:** J. Wise

**Affiliations:** Wiser Minds Ltd, Director, London, United Kingdom

**Keywords:** Secondary Gain

## Abstract

There is a wealth of data to tell us that, when it comes to illness, not all is as it seems. Research into hidden agendas of patients [1]drives home the point that a substantial portion of patients (up to 42%) have covert motives for obtaining secondary gains associated with their patient status (e.g., financial support, help or attention from others, stimulant medication, work or study related privileges, or evasion of responsibilities. Less than 10% shared their expectations with the psychiatrist. The Accident Compensation Scheme in New Zealand, , reported a prevalence of symptom exaggeration of 20-50%. In 2017 a disorder struck in Sweden. It struck whose families had failed their last appeal for asylum. The previously unknown ’catatonia’ has many of the characteristics of a culture bound syndrome – giving voice to the voiceless/powerless. Researchers from Ireland studied the motivations of people with factitious disorder. A desire for affection was the most commonly mentioned reason for fabricating illness and as a coping mechanism for threatening life events. The analysis showed that motivation was conscious. Bianchini et al have reported on the Financial Incentive Effect. Perhaps counterintuitively one of the most important points they make is that the presence of a financial incentive is associated with worse outcomes. They found that factors othere than the injury itself control for the probabilities of return to work. How can we determine what is real? [1] Van Egmond, J., Kummeling, I., & Balkom, T. A. (2005). Secondary gain as hidden motive for getting psychiatric treatment. European Psychiatry, 20(5-6), 416-421.

**Disclosure:**

No significant relationships.

